# Biographia Medica

**Published:** 1831

**Authors:** 


### II. Original Intelligence.

28. *Biographia Medica.*—We propose to unite in a short article, a sketch of the lives and character of two friends, (claimed as such by ourselves, and claimed by each other,) Dr. John D. Godman, and Dr. Robert Best. A full biogrophy is not intended, as much (not too much) has been already published of the former; and the latter has left behind him too few memorials of the genius with which he was signally endowed, to justify an extended notice.

Several reasons suggest to us the association of these names in one article. The deceased were physicians and naturalists; they were both (but especially the latter) in great degree destitute of early education; each was without the benefit of patrimony; each was apprenticed to a trade;—at which one of them labored for many years; early and deeply smitten with the love of nature, each forsook the workshop, and dedicated himself to the study of physical science; in 1821 they became acquainted, each discovered in the other a sympathy of feeling, an ardent friendship sprang up, and when separated, it was cherished by an affectionate correspondence; at length, each embraced the christian religion, and wrote to the other of the spiritualities which lie behind the veil of that nature which they had admired together; finally they both fell victims to a pulmonary disease, and descended into the 'valley and shadow of death' in the same year.

Of Dr. Goodman's early years, we have received a number of interesting memoranda, from his first medical preceptor, Dr. Luckey, now of Circleville, in this state. According to this gentleman, Dr. G. Was born at Wilmington, in the state of Delaware. At an early pe iod he lost his parents, and was left without patrimony, or deprived of it. Dr. Luckey first saw him in 1810, when he was 15 years old. The Dr. was, at that time, a senior student in the office of Dr. Thomas E. Bond, of Baltimore. "The office" says Dr. L., "was fitted up with taste, and boys, attracted by its appearance, would frequently drop in, to gaze on the labelled jars and drawers. Among them I discovered, one evening, an interesting lad, who was amusing himself with the manner in which his comrades pronounced the 'hard words,' with which the furniture was labelled. He appeared to be quite an adept in the Latin language. A strong curiosity soon prompted me to inquire "Who are you?" "Don't you recollect" says he, "that you visited a boy at Mr. Creery's, who had a severe attack of bilious colic?" "I do. But what is your name my little boy?" He was small of his age. "My name, sir, is John D. Godman." "Did you study the Latin language with Mr. Creery?" "No, he does not teach any but an English school." "Do you intend to prosecute your stu dies alone?" "I do. And I will, if I live, make myself a Latin, Greek, and French scholar."

In the autumn of 1811, Dr. Luckey commenced the practice of medicine in Elizabethtown, Pennsylvania, and the next summer received a letter from his *protogee*, stating that he had been bound an apprentice to the printer of a newspaper. With this business, he was, from the beginning, exceedingly dissatisfied, as he evinced in his numerous letters to Dr. Luckey.

In one of these, dated July 23d, 1812, he expressed the opinion, that it was worse than 'cramping his genius over a pestle and morter'—it was 'cramping it over a font of types, where there are words without ideas.'

Addicted to reading, and aspiring to a more intellectual pursuit, it is not probable that our young printer was much devoted to the drudgery of the office, or performed his duties *con*

*amore;* which may sufficiently explain the origin of the difficulties, set forth in the following paragraph from a subsequent letter to the same.

"Every thing is in *statu quo* with me. The same series of oppressions, impositions and insults are still my lot to bear. But I will not bear them long. From the oldest to the youngest, master and man, all seem to have a disposition to peck at me. You will (or may be) surprised to hear that I can never make a printer. It is an erroneous opinion of some people, that no one can be a printer unless he be a scholar. On the contrary, scholars can hardly, if at all, be printers. I would not wish you to think that I count myself a scholar. On the contrary I think myself no scholar."

The following extract from another letter, dated October 23d, 1813, shows that, at this early period, young Godman was threatened with the malady which ultimately destroyed him.

"The disease for which I mentioned a recipe in my last has commenced its direful effects on my poor body. A continued pain in my breast, and at night, a slow but burning fever, convince me that I am travelling down a much frequented road to the place where disease has no effect. This my friend is no phantasy. I do not say it from affectation. I feel it. I cannot believe in this disease being contagious, or I should be certain that I have caught it. I sleep with a youth who was born with it and has it fully."

In the opinion of Dr. L., the deceased, at that early period, labored under a hypertrophy of the heart.

Through the whole of his apprenticeship, young Godman had a strong desire to study medicine, but his guardian was opposed to any change of destination. Early in the month of January, 1814, he writes to Dr. L.—

"At the suggestion of Dr. Anderson, I have determined to commence the study of Chemistry, as he says it will be a great improvement to the mind, and more so, I may be enabled, the ensuing season (if I should live so long) to attend the Lectures at the University (of Maryland,) and it seems to run greatly in Dr. A's head that I shall one day be a physician. How far this surmise may be right, time will disclose. It may indeed so happen, and should I study Chemistry, now, I shall not have it to do at a future period. I must, however, ask your opinion in this affair."

On the 24th of the same month, he writes to the same gentlemen—

"I have read the catechetical part of Parke's Chemistry, and I can assure you I liked it not a little. But my knowledge, so far as I may obtain it, will only be theoretical."

In the same letter he sets forth his early views of the Christian religion:

"I have not ever had a fixed determination to read the works of that Modern Serpent,* nor had I determined *not* to do it; and it seems to me surprising, that a fellow student of yours should recommend the perusal of such writings as Thomas Paine's.

I had, thank Heaven, before I asked you the question, and still have, the "Apology for the Bible," by the celebrated Lord Regius, of Landaff. (Bishop Watson.) There is a great comfort in the belief of that glorious doctrine of salvation, that teaches us to look to the Great Salvator for happiness in a future life; and it has always been my earnest desire, and I must endeavor to die the death of the righteous, that my last end and future state may be like his. It would be a poor hope indeed—it would be a sandy fountain for the dying soul, to have no hope but such as might be derived from the works of Bolingbroke and Paine; and how rich the consolation and satisfaction afforded by the glorious tidings of the blessed Scriptures. It is my opinion, there has never one of these modern deists died, as their writings would lead us to believe; nor are but few of their writings read at the present day."

In the year 1814, when the war raged in the Chesapeake, he became a sailor under Com. Barney, and was engaged in the service at the bombardment of Fort McHenry. Early in the next year, Dr. Luckey, captivated by his genius, and touched by his misfortunes, resolved to invite him to his house, in Elizabethtown, and afford him all the facilities in his power for studying the profession to which he aspired. It does not appear how he had rid himself of his apprenticeship; but he seems to have been at liberty to accept the Doctor's generous invitation. This he did, with emotions of joy which are uttered in the following simple and affecting reply, dated April 4th, 1815.

"I have this hour received your last letter, and I can assure you, that language is inadequate to express to you my sincere, unfeigned joy, for the pleasing news you have communicated to me. Let the manner in which these lines are penned, convince you of the state of my

* Thomas Paine.

mind at present. I was, 30 minutes before I received your letter, on the point of going to a printer, in this city, to seek employment, and, but for Providence, I should have done so. You may suppose that, as soon as I read your letter, I abandoned this intention and returned to my sister's house,* "with fire in each eye and paper in each hand," to answer your epistle of friendship's own dictating. I must lay this aside for a short time, till my mind becomes settled and undisturbed. I stopped at the line above, in order that I might recover a small degree of composure, in order to express myself as I ought, to so good a friend. I will certainly comply with your request, should it please God to continue my health and strength during the ensuing week. Should it please the mercy of Providence to suffer me to take up my residence with you, I shall endeavor, by the most indefatigable study and diligence, to give you the satisfaction your kindness to me deserves. I am in hopes that I shall be able to come some day in the course of the next week; but, as my journey must be a pedestrian one, I should not wish to mention a particular day."

"On the 10th of April, four days after the date of this letter, he arrived" says Dr. L., "at my house, and took up his residence in my family. He made his promise good, for in *six weeks* he had acquired more knowledge in the different departments of medical science, than most students do in a year. During this short period he not only read Chaptal, Fourcroy, Chesselden, Murray, Brown, Cullen, Rush, Sydenham, Sharp, and Cooper, but wrote annotations on each, including critical remarks on the incongruities in their reasonings. He remained with me five months, and at the end of that time, you would have imagined from his conversation, that he was an Edinburgh graduate. When he sat down to study, so completely was he absorbed by his subject, that it seemed as though the amputation of one of his limbs would scarcely withdraw his attention."

A circumstance having no connection with the relation between him and his benefactor, but involving them both, led to premature separation. One or both of them were requested by the political party to which they belonged, to deliver orations on the approaching Fourth of July. Dr. L. began at the appointed hour, and went through with his discourse, but attempts were made by the opposite party to offer insult and create disturbance; at which our young orator became indignant;

* Mrs. Stella Miller, of Baltimore.

and, yielding to the impulse of his strong native feelings, not only refused to deliver what he had prepared, but resolved on returning forthwith to Baltimore. His oration was left with his preceptor, who speaks of it as not unworthy of Patrick Henry.

Departing from Elizabethtown, he returned to Baltimore, and became a pupil of Dr. Hall; and, in the succeeding autumn, began to attend the lectures in that city. His pecuniary difficulties, however, were pressing, and, in the ensuing February, 1816, he wrote to his benefactor, in the following eloquent and affecting style:

"Need I then inform you how high my expectations were raised, when I commenced attending the lectures this winter—Need I say I was almost certain of future competency? Alas! my friend, the Great Ruler of Events has interposed, (in order to teach me resignation to his will,) this heavy disappointment By unforeseen events—by domestic calamities, I have been compelled to relinquish the study of medicine, so long the ultimatum of all my hopes. FATHER OF ALL, THY WILL BE DONE. I have made this my motto—my consolation; and did I not daily see the truth of "*Omnia pro optimo*," I might perhaps repine. I am now in expectation of a situation with an eminent apothecary of this city, and I may be enabled, at a future period, to recommence the study of medicine."

This situation however he did not obtain,

"Let me now give you a retrospect of 'the days of my life.' Since I have returned from you, I have discovered my *real* age, in an old book of my father's, (and you would hardly suppose it,) I was 21 years old the 20th day of December, 1815. Before I was two years old I was motherless—before I was five years old I was fatherless and friendless—I have been cast among strangers—I have been deprived of property by *fraud*, that was mine by right–I have eaten the bread of misery—I have drunk of the cup of sorrow—I have passed the flower of my days in a state little better than slavery, and have arrived —at what? Manhood, poverty, and desolation. Heavenly Parent, teach me patience and resignation to thy will."

About this time he seems to have found a patron in Professor Davidge, and, on the 18th of April following, he wrote to Dr. Luckey—

"I still continue to study with Dr. Wright, (the partner of Dr. Davidge,) and provided it shall be the will of Heaven, I may possibly procure admission in the course of the next year into the venerable circle of Medicine."

In speaking of his perplexed and embarrassed situation, and of the mutations of fortune, he says—

"There is only one thing which points to, and affords immutable consolation, and that is, the observance of Religion. Although we should be incapable of reaping enjoyment in this world, even from uninterrupted prosperity, yet we can ardently long for, and sincerely believe, we may be eternally happy in the next."

In this situation he finished his medical situation. In the language of Professor Sewell*—

"Here he pursued his studies with such diligence and zeal, as to furnish, even at that early period, strong intimations of his future eminence. So indefatigable was he in the acquisition of knowledge, that he left no opportunity of advancement unimproved, and notwithstanding the deficiencies of his preparatory education, he pressed forward with an energy and perseverence, that enabled him not only to rival, but to surpass all his fellows."

He appears to have attended the lectures in the Baltimore school, through the sessions commencing in the autumns of 1816 and 1817. In the course of the last, Professor Davidge was disabled, by an accident, for several weeks, and Mr. Godman was appointed to supply his place. This, as he had been an apprentice to a trade, not three years before, in the same city, was an honorable testimony to his talents and industry, and must have been highly gratifying to his ambition. According to Professor Sewall, (*loco citato*)

"This situation he filled for several weeks with so much propriety—he lectured with such enthusiasm and eloquence, his illustrations were so clear and happy, as to gain universal applause; and at the time he was examined for his degree, the superiority of his mind, as well as the extent and accuracy of his knowledge were so apparent, that he was marked by the Professors of the University as one who was destined at some future period, to confer high honor upon the profession."

---

* Eulogy on Dr. Godman, p. 4.

In reference to his graduation, on the 10th of February, 1818, he wrote to his friend, Dr. Luckey, in these emphatical words:

"I know not what to tell you for news, unless I tell you that I passed my graduate examination, on Saturday; (Feb. 7,) which lasted 20 minutes; and, of course I have now the 'vast unbounded prospect all before me;' though 'shadows clouds and darkness rest upon it.' I will go to the country to practice, most probably to Frederick County."

In the United States, it is common to see young men, without preparatory education or fortune, become practitioners of medicine; but most of this class struggle into the ranks of the profession, totally unprepared; and depart from it for other pursuits, or for the grave, unknown and unhonored by the scientific world. *Such* an admission, must not be confounded with that of young Godman; who scorned to enter the profession unqualified and unauthorised by those who guard, or ought to guard, its portals. In this respect he was a shining example; and his subsequent success should animate every friendless young man, who may engage in the study of medicine, to imitate his industry and unfaultering perseverance. By these means, if not blessed with his genius, they may prepare themselves for extensive usefulness, and earn respectability if not renown.

We come now to contemplate Dr. Godman, as a member of the profession. His first location was in the village of New-Holland, on the banks of the Susquehannah; where, however, he remained but a few months. The next was on the Petapsco, near Baltimore, whence, in July, 1819, he wrote to Dr. Luckey as follows:

"My success in business has been considerable, or my practice, at least, has been as extensive as I could rationally expect."—"What my success may be in the end is at present very doubtful. I still have considerable expectation of being recalled to Baltimore, in order to fill the place which I held in the University. If it so happen, I shall be much delighted, as a country life is very little, or not at all to my taste."

In these rural situations he devoted himself to the study of nature; and, at a subsequent time, set forth the fruits of his observations in a series of papers entitled, the "Rambles of a Naturalist." But his ardent temperament was little adapted to the stagnant existence of a village doctor. He thirsted for competition, and longed to engage in the rivalries which prevail among the candidates for fame. Nature seems to have urged him on. It was she who revealed to him the compass of his intellectual powers; and bid him seek a theatre commensurate with their efficiency. A different arrangement from what he had anticipated was made in the Baltimore school; he returned, however to that city, but at length boldly resolved to fix himself in Philadelphia, and become a public teacher of anatomy and physiology.

But an unexpected event gave, for the time being, a different direction to his efforts. The writer of this article was inquiring, at that time, for a suitable person to fill the chair of Surgery in the Medical College of Ohio, the first session of which had just closed; and Dr. Godman was recommended. His qualifications for the first place, were expressed by Professor Gibson, then of the University of Pennsylvania, but previously a member of the Baltimore institution, in the following unequivocal and prophetic language. "In my opinion, Dr. Godman would do honor to any school in America." He was forthwith appointed; and arrived in Cincinnati the ensuing October, (1821,) in time to enter on the second session of the school.

For the practical details of such a professorship, he could not of course be well prepared, as his surgical experience was exceedingly limited; but he was learned in the institutes of the science, aud his knowledge of Anatomy was comprehensive, accurate and commanding. As a dissector, he was equally rapid and adroit. His lectures were well received by the class, who admired his genius, were captivated by his eloquence, and charmed with the *naivette* of his manners.

In the course of the session, difficulties of which he was neither the cause nor the victim, were generated in the faculty, the class was small, and the prospects of the institution overcast: under these circumstances, Dr. Godman resigned, but did not at that time return to the East.

Not long before, the author of this narrative had issued proposals for a medical journal, to be edited by the professors of the College, and obtained a number of subscribers; but the distracted state of the institution, prevented the fulfilment of the design. To this enterprise, as soon as he had resigned, Dr. Godman directed his attention, and, assisted by Mr. Foote, a liberal and literary bookseller in this city, in a few weeks issued the first number of the *Western Quarterly Reporter.* Thus, if not the first to project, Dr. G. had the honor of being the first to commence, a journal of medicine, in the Valley of the Mississippi. At the end of the 6th number, of a hundred pages each, the work was discontinued, for, previously to that time, its editor had returned to Philadelphia. More than 300 pages of this periodical were from his own pen; chiefly in translations and reviews of anatomy, Physiology, and Medical Jurisprudence.

Dr. Godman resided in our city for one year only; but in that short period he deeply inscribed himself on the public mind. The memory of his works still remains with us. In addition to writing for his medical journal, and to his practice, which was considerable for a stranger, he erected an apparatus for sulphurous fumigation, and translated and published a French pamphlet on that remedy; he read medical books, and many current works of general literature; prosecuted the study of the German and Spanish languages; and labelled the ancient coins and medals of the Western Museum. In the midst of the whole, he found time to cultivate his social relations; and every day added a new friend to the catalogue of those, who loved him for his simplicity and frankness, not less than they admired him for his genius, vivacity, and diligence. Thus, to use an idiomatic expression, he was a growing man, and might have remained with us and done well. But the hand of destiny was upon him. He had left the banks of the Petapsco, to be a public teacher: the same object had drawn him from Philadelphia to Cincinnati; and that object, at length, restored him to the great emporium of the medical sciences. Contrary to the wishes and importunities of his western friends, in the autumn of 1822, with his young family, he set off for the theatre of his future glory; which he reached in safety, though not without some of the many of the difficulties, at that time connected with a journey across the state of Ohio; of which, in a letter from Wheeling to one of his friends in this city, he gave a familiar account, in all respects so characteristic, that we hope to be excused for extracting it:

"We arrived last night, after a journey which exceeded in miseries any twenty journeys I ever made in my life. Thank God, the whole has been productive of nothing worse, than some hoarseness to my wife, and a galloping consumption of my bank notes. We were thirteen days on the way, *twelve* of which gave us as heavy rains as ever poor mortals could venture to travel in; and this produced such a delightfully *soft* state of the roads, that but for the rocks, (which fortunately were not twenty feet below the surface,) we might have been extracted some thousand years hence, in a high state of preservation, to decorate Best's museum, having one of Dorfeuille's mummy labels around our necks.

If I were one of the "tristful travellers," I might draw much "matter of melancholy" from these "misadventures," as my friend Sancho Panza calls them. But as the blessed sun of Heaven has driven forth once more in his beamy chariot, and the clouds are scattered from their long held seats, those which have loured on my mind, have also fled; and with "a light heart," I am once more preparing to encounter all the good or ill that God may send."

Of Dr. Godman's life and labors from this time forward, we shall say but little, as they are known to all the reading people of the United States, both in and out of the profession; and as our chief object is to present the difficulties and triumphs of his earlier years, for the benefit of our younger readers.

In Philadelphia he immediately began to lecture on anatomy and physiology, his first and greatest objects; and succeeded so well, that, in 1826, he was called to Rutgers' College, in the city of New-York, as an associate of Mott and Hossack.

In 1824 he was made one of the editors, (a *working* editor,) of the Philadelphia Journal of the Medical Sciences; and continued a liberal contributor to that respectable periodical, to the last weeks of his life.

At different times he published a number of interesting and eloquent introductory lectures.

He was the writer of several elaborate analytical and critical reviews, in the American Quarterly.

At the present time, actual discoveries in anatomy are no more to be expected, yet Dr. G., with admirable skill, revealed many new connections and relations of certain parts, and described them in a volume which he entitled Anatomical Investigations.

He translated and published from the Latin, French, and German languages, a variety of papers and distinct treatises; several of them on subjects not professional, as for example, Lavasseur's Narrative of La Fayette's Visit to the United States.

He wrote critical and emendary notes on several important English and continental works, which the booksellers of this country were about to publish.

The article Natural History, in the Encyclopedia Americana, was exclusively confided to him, and his labors upon it ended only with his life.

He studied the zoology of N. America, both existing and fossil, and favored us with an interesting and extended history of all its known quadrupeds, embracing a great variety of new observations.

Such were the labors of the deceased, during tha seven years that he resided in Philadelphia and New York. For the whole of that period, his life was one of unmitigated toil. As far back as November, 1823, he writes to his friend Dr. Best,

"Whatever you may think of my long continued silence, it has been unavoidably produced by the incessant and laborious employments which have occupied the whole of my time."

In 1824, he writes to another friend—

"My time has been very much occupied in the various duties which devolve on me here, and I am obliged to neglect my friends, in appearance, because it is out of my power to bestow the necessary attention to correspondence."

Again, in 1825, he says to the same—

"It is needless to tell you, that I am excessively occupied, and shall be more so as the winter approaches."

In the next year we find him still in the same condition—

"If you expect news at my hands," says he to Dr. Best, "you expect in vain. My life is one monotonous round of incessant toil after bread and *fame*, that '*certain* portion of uncertain paper.' Of my success in the bread making way, I can, thank God, speak more satisfactorily, than when we last met, though still nothing to boast of."

Again in the same year he writes—

"You recollect how much and how hard I had to work, when you were here—that was nothing to what I have to do now, as vigilance and labor are incessantly demanded, not only to gain more 'reputation,' but to retain that which I have already with vast toil acquired."

In the following year after he had removed to New-York, and was there a candidate for professional business, he writes to the same friend—

"The prospects of our college are fair enough at present, but what will be the event, cannot be told until the time of trial arrives. For my own part, I am not a little sick of the life such a business occasions, and think you far better off, in a situation, where you can acquire a subsistence and respect, without the incessant worry and vexation attendant on a life of professional ambition. For my own part, I shall lay myself as much out for the profession as I can, though I fear, not the best subject for improvement in that way. My situation is such, that I am obliged to rely, in a very great degree, on my pen, and that, you will say, produces habits very little compatible with the introduction of one's self into practice, where there are so many professed bowers, scrapers, and flatterers."

In the ensuing winter he was seized with the disease of the lungs, of which he finally died, and was compelled to suspend his lectures. In the following January, 1829, he speaks to the same gentleman, of his situation and labors, in these affecting words—

"My excessive exertion, and the exposure to a dreadful climate destroyed me. My lungs became diseased, and last winter, I was threatened with so rapid a decline as to force me to escape from the climate of New-York, by going to the West-Indies. The months of

February, March, and April, my wife and I spent in the Danish Island of Santa Cruz, where I very nearly perished from my disease, though I certainly should have done so in New-York. On my return to Philadelphia, in May, I took a house in Germantown, within seven miles from the city, where I have since resided. During the warm weather I was able to creep about, but since the first of the fall have been confined to a single room. My health during all this time has been in a very wretched state; and my *consumption* very obvious indeed, for I wasted to bones and lost all my strength. Until the last three weeeks past, I was exceedingly low, unable to sit up, eat, or perform any function advantageously. Since the time mentioned I have greatly recovered in all respects. My cough is by no means troublesome, and I eat and sleep well. What is best of all is that I have never had hectic since leaving New-York, where I was not properly prescribed for. Notwithstanding all these drawbacks, I have had my family to support, and have done so merely by my pen. This you may suppose severe enough for one in my condition, nevertheless necessity is a ruthless master. At present, that I am comparatively well, my literary occupations form my chief pleasure, and all the regret I experience is, that my strength is so inadequate to my wishes. Should my health remain as it is now I shall do very well, and I cannot but hope, since we have recently passed through a tremendous spell of cold weather, without my receiving any injury. All my prospects as a public teacher of anatomy are utterly destroyed, as I can never hope, nor would I venture if I could, again to resume my labors. My success promised to be very great, but it has pleased God that I should move in a different direction."

In the following year, continuing to write for the support of his family till the last month of his existence, he was taken from them, and in him they lost their all. Twelve years of unfaultering industry, that had carried his name into all the countries where science is cultivated, had not enabled him to accumulate property; and ended by consigning him to the grave, ere he reached the noon-day of life, or had put forth, to their full extent, the vast intellectual powers, with which he was endowed. In all this, there is much more to grieve than astonish us. As a physician and surgeon, Dr. Godman's business was never considerable. At the very beginning of his professional career, his mind took a different direction. No human heart was ever imbued with a deeper thirst for knowledge, or warmed with a nobler love of glory. He made the former subservient to the latter; but the objects of his ambition were teaching and writing, not the practice of his profession. Perhaps, indeed, he adapted the aims of his ambition to his taste. He relished reading, writing, and lecturing, more than the practice of medicine; and sought to derive from them, that emolument, which, in this country, they seldom afford, and which can much more certainly, be drawn from a close attention to the practical duties of the profession. Had he possessed a patrimony, this course would have been unexceptionable: without such a reliance, no young physician should neglect the means of acquiring professional business, at the outset of his career.

Dr. Godman was, without doubt, a man of genius; but he was not, perhaps, so much the expositor, as the historian of nature. Observing, imaginative, fluent, and graphical, he abounded, less in deep and original analyses, than vivid and accurate delineations. Thus his mind, like that of Lucretius, Darwin, and Good, was poetical and philosophical; and he left him several fugitive pieces, written chiefly in his last illness, which prove that he might have shone as the poet of nature, not less than her historian, had circumstances awakened his powers.

He possessed uncommon abilities for dissection, and was accustomed, in the presence of his class, to disentangle the structures intended for exhibition; thus showing their connexions and dependences, while he described them with that clearness, animation, and eloquence, which only can render the study of anatomy attractive.

In every situation, and on every subject, his attention was active and acute, his perceptions rapid, his memory exceedingly retentive, and his rationation, profound and analytical.

For languages, he had both taste and talents; and, succeeded in acquiring a practical knowledge of a greater number, perhaps, than any American physician who had preceded him.

The qualities of his heart harmonized with those of his head. They did honor to the profession, and inspired confidence wherever he went. To pure moral habits, and incorruptible honesty, he added that unsuspecting frankness, and all those fine and glowing sensibilities, which at once excite our respect, and win our affection.

But it is not our design to attempt an extended delineation of his character, and we shall close an article already prolonged far beyond our original intention, with his own statement of his opinions and hopes, in regard to that world of which he is now a 'bright inhabitant.'

In his last letter to Dr. Best, who followed him in a few months, he writes:—

"It gives me great happiness to learn, that *you* have been taught, as well as myself, to fly to the Rock of Ages for shelter against the afflictions of this life, and for hopes of eternal salvation. But for the hopes afforded me, by an humble reliance on the all-sufficient atonement of our blessed Redeemer, I should have been the most wretched of men. But I trust, that the afflictions I have endured, have been sanctified to my awakening, and to the regeneration of my heart and life. May we, my dear friend, persist to cling to the only sure support against all that is evil in life, and all that is fearful in death."

Thus fell, from the firmament of the American profession, before he had reached his meridian splendor, one of the brightest stars which have yet risen above its horizon; but he was one only, and, we may hope, that his own example will contribute to place some other in the constellation.

---

We have devoted so great a space, to extracts from the letters of Dr. Godman, that our notice of his friend, D[illegible]. Best must be condensed; but this we regret the less, because, in Dr. Best, we behold the existence of great powers, rather than the attainment of great distinction. Such an one may be admired by his friends, but they have no right to exact the admiration of others. We do not, however, admit, that the object of our own affectionate respect, has no claims upon that of the profession, in this country. On the contrary, we see, in his history, not a little that deserves to be recorded for the information of others; who, placed under circumstances far more auspicious, too often accomplish far less. Indeed, we shall take the liberty of saying, that, throughout the United States, a *majority* of the profession, seem to have few aspirations after fame; no *quenchless* love of science; and, but small regard for the interests and dignity of the honorable calling, to which they belong. It is cheering, therefore, to meet with men of genius, who rightly appreciate its character, acknowledge its claims, and, conscientiously, devote themselves to the means of advancing its reputation. Of this little band, Dr. Best was one, who, in the fullness of years, would have become a bright and beautiful model.

He was a native of Somersetshire, England, and was brought to the United States, when 12 years of age. About the year 1803, his father settled in this town. Of his childhood, we know but a single fact, which is, that he never went to school but three months. His father, a man of strong and philosophic intellect, was poor, and had several sons, whom he brought up to his own trade—that of a watch and clock maker. Our first knowledge of Robert, the subject of this notice, dates back to the time of his apprenticeship, when he devoted all his leisure hours to the study of Chambers' Cyclopædia, and a few old books of Natural Philosophy, Anatomy, and Natural History, which his father had, at different times, picked up for his own perusal.

In due time, he became a master workman, and established himself among us as a candidate for business. As a silversmith, (having added that business to the other,) he was distinguished for taste, and an accurate and comprehesive knowledge of every chemical art, connected with *Metallurgy.* As a watch-maker, he was equally remarkable for his acquaintance with the principles of *Horology;* like Rittenhouse, having scientifically studied the mechanism of clocks, watches, and chronometers, which, the majority of artists, look at only with the eye of empyricism. But his skill was not limited to instruments of this kind. It extended to those which are employed in all the experimental sciences; and his studies and labors upon these, not unfrequently withdrew his attention from the current details of his trade, and abridged its productiveness.

In all this, his friends saw a love and power for scientific knowledge, which made them feel sorry that he had not been born under circumstances more congenial to his taste, and propitious to his genius. At length an opportunity for transplanting him arose, and he was placed in a new situation.

In the year 1818, a number of public spirited citizens projected the Western Museum, now one of the brightest ornaments of our city; and Mr. Best was appointed its Curator and Artist. It was his duty to preserve, arrange, and label the various productions of Nature and Art, and no employment could have given him greater delight. He applied himself, anew, to the study of the sciences involved in this labor; and soon manifested attainments corresponding, in some degree, with his skill as a maniputator. In the depths of winter he visited the Indian country, and returned with the skins of many of its rarest animals, among the rest of the panther, which Dr. Godman has figured in his Natural History, where he has also published a long letter from Mr. Best, on the habits of that animal. He explored our fields and forests and streams, and brought home specimens of their inhabitants, which he preserved, and "set" with a degree of taste and skill, which we have not seen equalled in the United States; while he embellished the cases in which they were enclosed, with landscapes, which proved him a painter of strong and original powers.

The establishment being, at length, opened for public exhibition, and arrangements made for instruction by lectures, Mr. Best was called upon to teach, and in the autumn of 1820, delivered a course of experimental lectures on Electricity, which did him much credit. About the same time, he was appointed assistant to the professor of chemistry, in the Medical College of Ohio; and throughout the first session of the institution rendered important aid to that chair. But the class of the college was small, and the funds of the Museum society, too limited to afford him the requisite salary; and being offered the place of lecturer on Pharmaceutic Chemistry, in Transylvania University, at Lexington, he resolved, in the spring of 1823, to remove to that town; and, as connected with his official duties, to undertake the management of a chemical laboratory and warehouse, for drugs and medicines. Meanwhile, he had determined on making himself a physician; and, in addition to occasional readings, in the hours not otherwise appropriated, attended many of the lectures, and was thus growing every day in professional knowledge. His own lectures were distinguished for clearness and accuracy; his views were deep and analytical; and he called upon his pupils to descend with him into the depths of the science. To aid them, he compiled, during his second course, a number of tables; to which he prefixed a discourse, abounding in evidences of an intimate acquaintance, with some of the most recondite subjects of chemistry; and published the whole, under the title, of *Tables of Chemical Equivalents, Incompatible Substances, and Poisons, and Antidotes, with an Explanatory Introduction.* This was his only publication.

In the year 1825, he applied himself more diligently to the study of medicine, becoming, in the winter, a student of practical Anatomy, which he prosecuted with a degree of success, that we have never seen equalled; and which led to the remark, by his friends, that his talent for Anatomy was still greater than for Chemistry. During this winter, in conjunction with a student of the University, he repeated most of the experiments previously made by Magendie, and Coates and Lawrence, on the absorption of active substances into the circulation, and verified their conclusions; as we have stated in the first number of this Journal. In the spring of 1826, at the age of 34, he was graduated, and immediately commenced the practice of physic and surgery.

He was not slow in acquiring public confidence. Society had marked him as a man of the first order of talents, and his bland and simple manners, his great humanity, and his devotion to the sick, gave him, from the beginning, a practice which rapidly increased till the commencement of his last illness. As a surgeon, he was dexterous in the highest degree; as a physician, few that we have ever known, at any period of their lives, possessed a more admirable sagacity. It was impossible not to believe, that nature had destined him for great eminence in the practice of the profession; and had he lived, the zeal and genius, which, in despite of poverty, in the absence of all early education, and unassisted by powerful friends, had, in a few years, at middle life, raised him from the workshop, to merited and equal association with the most distinguished physicians of the region where he resided, must, ultimately, have enabled him to enlarge the boundaries of our science.

As it is, we can only present him, in connection with his friend Godman, as another evidence, that in this country, no disadvantages of birth or fortune, ought to be plead in justification of inferiority. To ascend from the humble walks, to the high places of society; from the work shop to the lecturer's desk; from the companionship of the unlettered, to an equal association with men of science, and substitute *their* admiration for the applause of the ignorant, requires, it is true, the genius, industry, and high aspiration of the two friends whom we deplore; but those who have one talent only, should not bury it in the earth and go to sleep, much less those who are more liberally endowed, and seem destined, therefore, to do some good in the world. As far as we have seen, professional inferiority is, much oftener the consequence, of defective application, and deficient ambition, than of sparing intellectual endowment. We must admit, however, the discouraging fidelity of the poet, who says,

"*Slow* rises worth by poverty depressed."

Nevertheless, let him who has adventurously put his hand to the plough, not look back, or fall by the way side. Though fame's proud temple may literally shine *afar* to him, each hour of labor brings him nearer to its gates: Like the ascent of the sun, his rise may be too slow to be noted; but whenever he works he wins his way.

For many years, Dr. Best suffered severely from bad health; his sensibilities were morbidly acute, and his temperament melancholy; soon after he had dedicated himself to the study of science, his only son, a child of uncommon genius, was drowned in the Ohio, and he lost a daughter of almost equal promise: thus, it might be said, that he prosecuted his studies "in sickness, and in sorrow." Through most of the dark transition from a lower to a higher orbit, he was an avowed disbeliever in the Christian religion; and of course unsustained by its consolations. Nearly three years before his death, however, he was induced to enter on an examination of that system, and the result was most happy, he admitted the validity of its evidences, and exhorted his scientific friends not to reject it without inquiry. He adopted its precepts, and opened his heart to its benign solaces; thus he superadded the Christian to the philosopher, and in his 40th year, yielded up his spirit in hope of peace. D.

---

29. *Professor Caldwell's Prize Essay on the comparative Influence of vegetable and animal decomposition in the production of Fever.*

We have read with great pleasure, the above essay. So much of the Journal has lately been devoted to the subject of marsh miasm mata, that we do not feel at liberty to publish any of his remarks upon this subject. We have the less regret upon this account, since the views of the learned author coincide very generally with those which have been advanced in former numbers of this journal. We can therefore take no further notice of this production, than simply to give a brief abstract of the distinction drawn by the author between intermittent and typhus fever.

1. The former is the product of changes in vegetable matter, and is generated where no animal matter can be found. Typhus, on the contrary, springs from animal matter, generally from changes that take place in the secretions of the human body, and is produced in situations where there is no decompo sable matter but human exhalations.

2. The two diseases differ in their access, type, progress, symptoms, and duration, having no other resemblance in these respects, than the general one of their common febrile character.

3. They are produced in very different situations. The origin of intermittents is, in the open air; and it is of little consequence in its production, whether the air be calm or agitated by winds. Typhus requires close and unventilated rooms, and a stagnant atmosphere.

4. Hot climates and the warm seasons of the year, are favorable to the various forms of intermittent and remittent disease.

Typhus is a disease of cold climates, and of the winter season.

5. The miasm which produces typhus, may adhere to articles of clothing, and thus be transferred from place to place. There is no reason to believe this to be the case with the causes of intermittent fever.

6. The miasm of typhus cannot be generated in the open air, nor transported to any distance by the atmosphere, without losing its deleterious character. Marsh miasm, on the contrary, may pass to a considerable distance, and still retain its virulence.

Marsh miasm is said to produce disease in domestic animals.

The same is true of typhus, but the disease in this case is produced by the exhalations of animals themselves.

Typhus, the author remarks, is produced from local sources, and although it is produced most readily, and is marked with most malignity, when the general constitution of the atmosphere is unfavorable to health, yet it may take place when nothing of the kind exists. All febrile diseases possess a specific character, and, although in their advanced stages they are said to become typhoid, nothing more can be meant than, that some symptoms have supervened that resemble typhus.

The origin of epidemic typhus, the author believes to be atmospherical. It differs from the origin of intermittent fever, in having no dependence upon place, season, or any of the sensible properties of the atmosphere. F.

